# Multiple primary malignancies of the liver and the colon: a complex diagnostic and decisional process with a final unanswered question

**DOI:** 10.1186/1477-7819-12-75

**Published:** 2014-03-29

**Authors:** Nazario Portolani, Gianluca Baiocchi, Carla Baronchelli, Federico Gheza, Stefano Maria Giulini

**Affiliations:** 1 Department of Medical and Surgical Sciences, Surgical Clinic, Brescia University, III Chirurgia, Spedali Civili di Brescia, P.le Spedali Civili, 1, 25123 Brescia, Italy; 2 1st Department of Pathology, Brescia Civil Hospital, Brescia, Italy

**Keywords:** Gallbladder carcinoma, Synchronous tumors, Cholangiocarcinoma

## Abstract

We herein present the case of a 78-year-old man with an incidental finding of a solid hepatic mass without symptoms and only a laparotomic cholecystectomy for acute cholecystitis in the past surgical history. A colonoscopy, a magnetic resonance imaging scan, a positron emission tomography scan, and a computed tomography scan completed the preoperative workup: a neoplastic lesion 4.3 × 3 cm in size was diagnosed at segments IV and V, associated with a neoplastic involvement of the splenic flexure without signs of colonic occlusion. After colonic resection, a frozen section on a granulomatous-like tissue at gastric border suggested a diagnosis of an adenocarcinoma of bilio-pancreatic type, changing the surgical strategy to include gastric resection and hepatic pedicle node dissection. The discussion turns around the idea that a final diagnosis of colon cancer with regional nodal involvement (pT3N1) and metastatic gallbladder cancer with multiple peritoneal seedings cannot be excluded.

## Background

Preoperative distinction between primary or metastatic cancer has to be as accurate as possible for hepatic lesions, because the treatment is generally different.

CT and MRI are considered the best imaging techniques for cancer staging and surgical strategy guiding; they can also lead to a strong suspicion of hepatocellular carcinoma, that is, through the vascular pattern evaluation, suggesting to go straight to surgery [1]. When a certain preoperative differential diagnosis between colonic metastasis and ICC is not possible, further investigations are mandatory.

## Case presentation

A 78-year-old man was admitted to our ward for the incidental finding at the ultrasound of a solid mass in the liver, without symptoms. His past surgical history consisted of only a laparotomic cholecystectomy for acute cholecystitis, with a liver biopsy diagnostic for aggressive chronic hepatitis (the patient was HBV and anti HCV negative). The patient was in good general condition, but significant morbidity was noted: chronic renal failure (creatinine value 1.4 mg/dl), chronic obstructive pulmonary disease, hypertension, and a recent asymptomatic myocardial infarction. The liver function was normal. At the computed tomography (CT) scan, a 4.3 × 3 cm neoplastic lesion was found in segments IV and V. There was no macroscopic involvement of portal tree and hepatic artery. Magnetic resonance imaging (MRI) showed the main lesion with a cystic or necrotic central portion and a peripheral enhancement with late pooling; a little satellite nodule was also discovered. Imaging was suggestive either for a peripheral intrahepatic cholangiocarcinoma or for a liver metastasis from an occult cancer, probably arising from the gastroenteric tract. Tumor markers seemed to support this last hypothesis: CEA, 30 ng/mL; Ca, 19-9 > 12,000 U/mL; and alpha phetoprotein 3 UI/mL, strongly suggestive for a cancer arising from a secretory epithelium. An 18 F-FDG PET scan showed captation in the previously described sites (liver and colon), without any other visible spots. Colonoscopy showed a neoplastic stenosis in the transverse colon, limiting the direct visualization of the right colon. A CT colonoscopy confirmed the neoplastic involvement of the splenic flexure without signs of colonic occlusion and our endoscopic biopsy was positive for adenocarcinoma.

With a preoperative diagnosis of colon cancer with synchronous liver metastasis, the clinical case was evaluated by a multidisciplinary team. The age of the patient, the chronic liver failure, and the recent acute coronary syndrome were considered by the oncologist as contraindications to chemotherapy, neoadjuvant nor palliative. A further cardiologic evaluation showed no contraction of the infero-posterior part of the myocardium with a slight reduction of the ejection fraction (40%). Myocardial scintigraphy showed a stable hypoperfusion in the corresponding zone and a limited reversible hypoperfusion at the inferior portion of the septum; the dipiridamol test was negative for ischemic signs and symptoms. The patient was considered by the cardiologist at low to medium risk of a severe ischemic accident in the postoperative period. The anesthesiologist defined an increased risk for surgery with an ASA score of 3 (American Society of Anesthesiologists’ score).

A synchronous segmental colonic and liver resection was planned.

The intraoperative evaluation confirmed the splenic flexure neoplasia with a single hepatic lesion, lying in the gallbladder fossa with an extensive gastric adhesion. No ascites and no macroscopic nodal involvement were evident. Intraoperative ultrasound was negative for further hepatic lesions.

**Figure 1:**
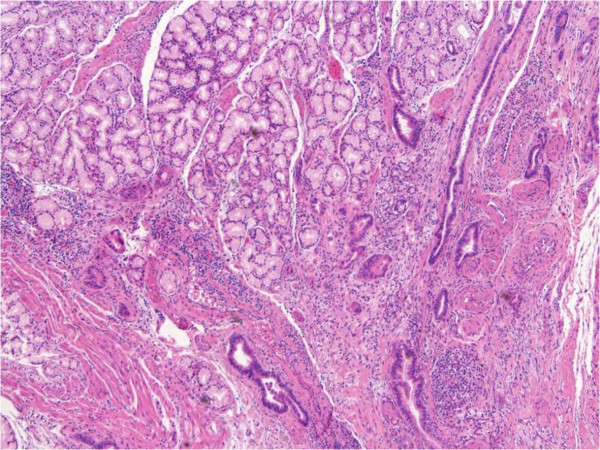
**Gastric border frozen section.** Low magnification hematoxylin and eosin frozen section on the granulomatous like tissue at gastric border.

**Figure 2:**
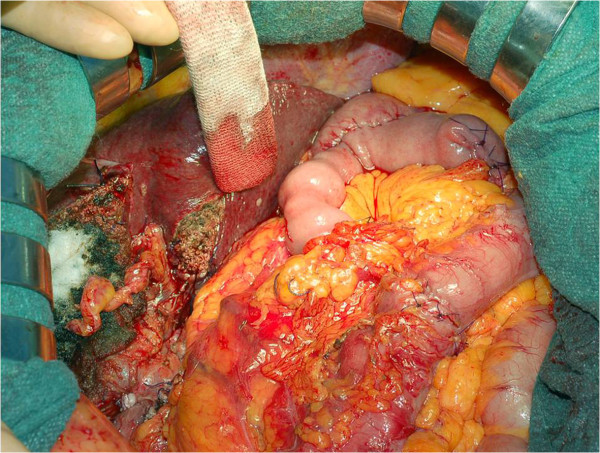
**Intraoperative view*. ***Intraoperative view at the end of the multivisceral resection and reconstruction.

First, we performed the resection of the splenic flexure of the colon with a regional lymphadenectomy, postponing the anastomosis after the liver resection. During dissection of the stomach out of the liver, a small necrotic cavity was opened with the appearance of several free biliary stones along the hepatic pedicle. The frozen biopsy of the residual granulomatous-like tissue at the gastric border was positive for *ab extrinseco* infiltration of adenocarcinoma, with a deep involvement up to the submucosal layer. The tumor showed duct-like structures lined by cuboidal cells. A cribriform pattern was present and there was heterogeneity of the neoplastic epithelial cells within the same gland together with the lack of tall columnar cells of intestinal type adenocarcinoma and lack of necrosis in the glandular lumina. Stroma was abundant and desmoplastic. These features were suggestive for a bilio-pancreatic adenocarcinoma instead of an intestinal one (Figure 1). As a result, the diagnosis was changed into multiple synchronous primitive neoplasms: intrahepatic cholangiocarcinoma (ICC) and colonic cancer. This unexpected finding required a more complex surgical procedure, including gastric resection and hepatic pedicle lymphadenectomy. The hypothesis of a curative procedure (no other localizations were evident) and the absence of any otherwise therapy (the patient was unfit for the chemotherapy) forced towards a radical, extended surgery. We proceeded with a liver bisegmentectomy (segments IV and V), hepatic pedicle lymphadenectomy, distal gastric resection, colonic anastomosis, and omentectomy. A peritoneal pelvic sampling was also performed (Figure 2).

On postoperative day 2 an asymptomatic myocardial infarction occurred, requiring a short recovery in the Intensive Coronary Care Unit of without any specific therapeutic care other than endovenous heparin. No surgical complications were recorded. The patient was discharged on postoperative day 20.

**Figure 3:**
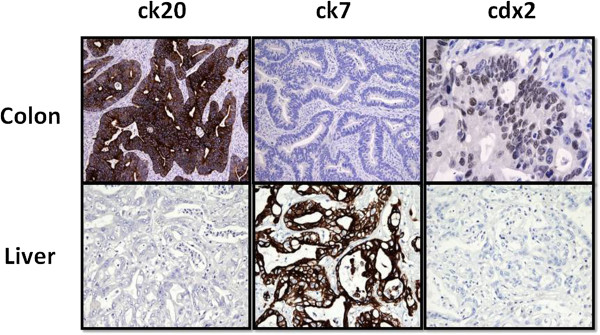
**Immunohistochemical analysis.** Final immunohistochemical analysis on surgical specimen. Ck20 positive, ck7 negative, and cdx2 positive for the colonic cancer, and ck20 negative, ck7 positive, and cdx2 negative for the liver. Ck20: dilution 1:50; Ck7: dilution 1:100; Cdx2: dilution 1:80.

**Figure 4:**
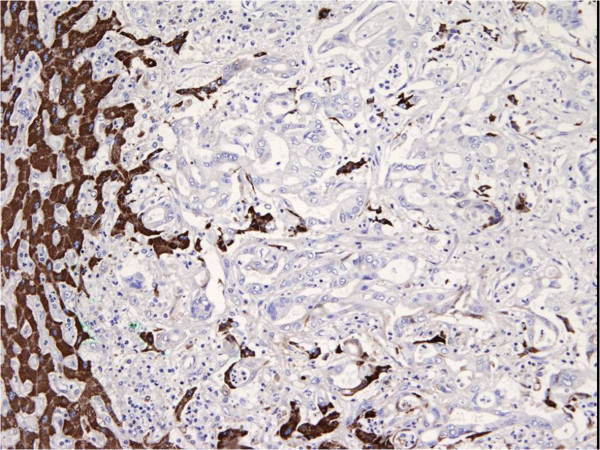
**Liver specimen pathology.** Final pathology on liver cancer specimen. The anti-hepatocyte monoclonal antibodies were negative.

**Figure 5:**
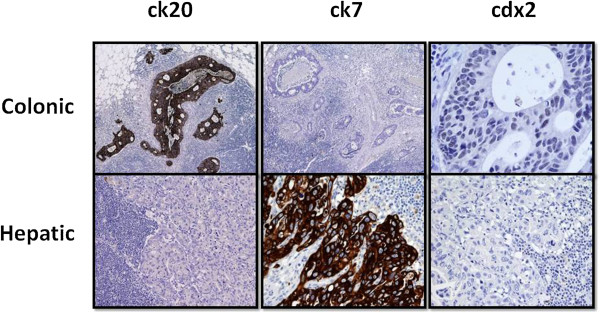
**Metastatic nodes.** Metastatic lymph nodes immonuhistochemistry: colonic type pattern for the metastatic nodes along the mesocolon and biliary profile for the nodes at the level of the hepatic pedicle and gastric wall.

The histological evaluation confirmed two distinct tumors: (1) adenocarcinoma of the colon infiltrating the adipose tissue, G2, with 3/16 metastatic nodes (Dukes C, Astler–Coller C2); and (2) adenocarcinoma of the biliary ducts with a central colliquative necrosis, a perineural, vascular, and lymphatic invasion and the metastatic involvement in five out of 10 perihepatic nodes and in four out of 10 perigastric nodes. The hematoxylin-eosin evaluation was suggestive for this diagnosis, but above all the complete panel of monoclonal antibodies. Immunohistochemical analysis with cytokeratin 20 (ck 20, *clone ks 20.8*, *Novocastra Menarini*), ck 7 (*clone ov-tl 12/30*, *Dako*), and cdx2 (*clone AMT 28*, *Novocastra Menarini*) defined two distinct profiles of positivity: ck20 positive, ck7 negative, and cdx2 positive for the colonic cancer, and ck20 negative, ck7 positive, and cdx2 negative for the liver (Figure 3). The anti-hepatocyte monoclonal antibodies tested on the liver cancer were negative (Figure 4). The immunohistochemical panel applied to the metastatic lymph nodes showed the same results: ‘colonic’ positivity for the metastatic nodes along the mesocolon and ‘biliary’ profile for the hepatic pedicle and perigastric nodes (Figure 5). Several unexpected small metastatic lesion of biliary origin were discovered in omentum and perigastric tissue; pelvic peritoneum was also positive for neoplastic involvement.

At this point, we reconsidered the past surgical history (urgent cholecystectomy 4 years before): the operating report described a difficult surgery, the gallbladder being removed after a wide opening of the wall. A pathology re-evaluation confirmed the initial diagnosis of acute necrotic ulcerative cholecystitis with slight dysplasia.

Three months after surgery, a diffuse abdominal recurrence (liver, spleen, adrenal gland, and mesenteric nodes) was noted. The patient died 9 months after surgery.

## Conclusion

For a differential diagnosis, two different strategies are feasible: exclusion of a gastroenteric origin of the tumor by colonscopy and gastroscopy, or doing a percutaneous biopsy (PB). For PB, in addition to technical problems due to reaching the lesion (this was not our case) and the specific risks of the procedure, some diagnostic difficulties can arise for the pathologist, given that different lesions share similar histological aspects. Therefore, an endoscopic study of the gastroenteric tract is considered the main diagnostic tool and when a primary colonic cancer is discovered, the diagnosis is generally considered certain (colonic cancer with a synchronous liver metastasis), and PB is usually not performed.

The following step is the definition of a therapeutic strategy, either immediate surgery (synchronous colonic and liver resection) or sequential therapy, where colonic resection, liver resection, and chemotherapy are differently combined in every single patient.

However, multiple synchronous neoplasms cannot be excluded *a priori:* colonic and biliary cancer association is rare but previously described [2] and carries some diagnostic and therapeutic problems. The first one, as above mentioned, is the difficulty to get a certain preoperative diagnosis, because the presence of a secretory epithelium histology does not allow you to differentiate the colonic mucosa from the biliary tract [3]. Immunohistochemical tests can help for differential diagnosis, but the global accuracy of this method (expensive and time-consuming) is not complete when applied on small specimens. Intraoperative evaluation also has some limitations, that is, considering that ICC and colonic metastases can be macroscopically similar, mostly due to the high percentage of fibrosis. In our case, only the intraoperative frozen examination allowed us making the diagnosis of multiple primary neoplasms; the *ab extrinseco* neoplastic involvement of the gastric wall (with normal mucosa) and the adhesion to the liver suggested an invasion by continuity from a primary liver cancer. This diagnosis changed our surgical strategy: ICC in our current practice requires hepatic pedicle node dissection, in particular for lesions larger than 5 cm [3]; according to neoplastic finding on gastric wall, a gastric resection instead of simple adhesiolysis was mandatory.

During gastric dissection, some free biliary stones showed up and this was the clear demonstration that the gallbladder had been violated during the previous cholecistectomy. This finding gives way to an intriguing hypothesis: the ‘biliary’ cancer may have been a gallbladder cancer and not a peripheral intrahepatic cholangiocarcinoma. In favor of this diagnosis the cancer was located in the gallbladder fossa and macroscopically the adhesion with the stomach seemed not to be a direct infiltrative growth of the tumor but a kind of easily fragmentable tissue with neoplastic involvement, as one can expect for an initially adhesive process with a subsequent neoplastic colonization. Furthermore, multiple micro-localization spread (perigastric, omentum, and pelvic peritoneum) would be expected after the dissemination of cancer cells and an iatrogenic perforation of the gallbladder is a well described possible etiologic mechanism, given that this is the principal concern to a laparoscopic approach when a significant risk of malignancy is supposed [4,5]. On the other hand, the long period of time between cholecystectomy and our intervention (almost 4 years) and the negativity of the pathology are against this hypothesis.

Gallbladder cancer is an interesting model of a step-by-step process with fixed progression time: after a first phase of metaplasia and low dysplasia, the evidence of an early cancer takes generally more than 12 years to show, while the passage to an advanced cancer takes only about 2 years; at this time the natural history of cancer is very quick, with a mean life expectancy of 3 to 6 months [6]. In the majority of cases, the process is clinically silent. Most gallbladder carcinomas originate in the mucosa as *de novo* carcinoma and the adenoma-carcinoma sequence has little importance in the histogenesis of this kind of cancer [7].

In the presence of an early stage cancer, histology is not rarely considered negative by expert pathologists [7,8]. Diagnostic dilemmas are particularly evident when the inflammatory alterations are prevalent; furthermore, usually in these situations no exhaustive evaluations of the specimen are performed, because without a clear cancer only two to three suspicious samplings from the different portions of the gallbladder are routinely done. Finally, the rapid re-growth of cancer with a diffuse metastatization pattern supports the hypothesis of gallbladder cancer. According to these considerations, a final diagnosis of colon cancer with regional nodal involvement (pT3N1) and metastatic gallbladder cancer with multiple peritoneal seedings cannot be excluded.

## Consent

Written informed consent was obtained from the patient for publication of this paper. A copy of the written consent is available for review by the Editor-in-Chief of this journal.

## Competing interests

The authors declare that they have no competing interests.

## Authors’ contributions

NP and CB conceived the case report and drafted the manuscript. BG and FG contributed to get the background, figures, and references, and helped to draft the manuscript. SMG revised the manuscript critically for important intellectual content. All authors read and approved the final manuscript.
